# Oleandrin: A Systematic Review of its Natural Sources, Structural Properties, Detection Methods, Pharmacokinetics and Toxicology

**DOI:** 10.3389/fphar.2022.822726

**Published:** 2022-02-21

**Authors:** Jinxiao Zhai, Xiaoru Dong, Fenglian Yan, Hongsong Guo, Jinling Yang

**Affiliations:** ^1^ Institute of Forensic Medicine and Laboratory Medicine, Jining Medical University, Jining, China; ^2^ Department of Forensic Medicine, School of Basic Medical Sciences, Fudan University, Shanghai, China; ^3^ Institute of Immunology and Molecular Medicine, Jining Medical University, Jining, China

**Keywords:** oleandrin, pharmacokinetic, oleandrigenin, enterohepatic circulation, toxicology

## Abstract

Oleandrin is a highly lipid-soluble cardiac glycoside isolated from the plant *Nerium oleander* (Apocynaceae) and is used as a traditional herbal medicine due to its excellent pharmacological properties. It is widely applied for various disease treatments, such as congestive heart failure. Recently, oleandrin has attracted widespread attention due to its extensive anti-cancer and novel anti-viral effects. However, oleandrin has a narrow therapeutic window and exhibits various toxicities, especially typical cardiotoxicity, which is often fatal. This severe toxicity and low polarity have significantly hindered its application in the clinic. This review describes natural sources, structural properties, and detection methods of oleandrin. Based on reported poisoning cases and sporadic animal experiments, the pharmacokinetic characteristics of oleandrin are summarized, so as to infer some possible phenomena, such as enterohepatic circulation. Moreover, the relevant factors affecting the pharmacokinetics of oleandrin are analyzed, and some research approaches that may ameliorate the pharmacokinetic behavior of oleandrin are proposed. With the toxicology of oleandrin being thoroughly reviewed, the development of safe clinical applications of oleandrin may be possible given potential research strategies to decrease toxicity.

## 1 Introduction

*Nerium oleander*, a commonly cultivated ornamental shrub of the Dogbane family, Apocynaceae (Bandara et al., 2010), known as oleander, is well-known for its extensive pharmacological activities and acute toxicity (Dey, 2020). Varieties of oleander are widely distributed in tropical and subtropical regions, and it is native to the Mediterranean regions of Africa and Europe (Shepherd, 2004; Bandara et al., 2010). Oleander is also planted in various provinces and cities in China and included in the Scientific Database of China Plant Species (Fang, 2013). Oleandrin is a lipid-soluble cardiac glycoside (CG) mainly obtained from *N. oleander*; it is the major toxic and pharmacologically-active constituent in all parts of the plant (Karawya et al., 1973; Langford and Boor, 1996).

Oleandrin plays an indispensable role in traditional medicinal practices globally as a highly potent herb with a wide spectrum of beneficial biological and pharmacological activities (Botelho et al., 2017). Oleandrin is of medical and toxicological interest, often used in folk medicine to treat various diseases, including congestive heart failure, abscesses, asthma, dysmenorrhea, sores, eczema, epilepsy, herpes, leprosy, malaria, ringworm, scabies, indigestion, strokes, and neurodegenerative diseases (Langford and Boor, 1996; Wang et al., 2000; Khan et al., 2010; Babula et al., 2013; Slingerland et al., 2013; Hong et al., 2014; Van Kanegan et al., 2014; Van Kanegan et al., 2016; Farooqui and Tyagi, 2018; Kanwal et al., 2020). Recent studies have highlighted that oleandrin also possesses favorable anti-tumor and anti-viral properties (Nasu et al., 2002; Afaq et al., 2004; Calderon-Montano et al., 2013; Singh et al., 2013; Botelho et al., 2020; Kanwal et al., 2020; Plante et al., 2020; Roth et al., 2020; Plante et al., 2021), including those against “enveloped” viruses. A recent study has shown that oleandrin is also effective against severe acute respiratory syndrome coronavirus 2 (SARS-CoV-2) infection (Plante et al., 2021). The oleander extract drugs PBI-05204 and Anvirzel, which contain oleandrin as the active principle ingredient, are in Phase I and Phase II clinical trials of malignant diseases (Roth et al., 2020; Terzioglu-Usak et al., 2020). Therefore, oleandrin has received a lot of attention from researchers and is now a primary focus of the pharmaceutical industry. However, oleandrin has a narrow therapeutic window, a complex pharmacokinetics profile, and is prone to inducing poisoning (Azzalini et al., 2019), which can result in arrhythmias, neurological disturbances, and even death. Related pre-clinical and clinical cases of intentional, accidental, and suicidal oleander consumption are frequently reported worldwide (Arao, 2002; Lin and Rong, 2007; Kozikowski et al., 2009; Al et al., 2010; Senthilkumaran et al., 2011; Kucukdurmaz et al., 2012; Papi et al., 2012; Renier et al., 2013; Bavunoglu et al., 2016; Azzalini et al., 2019). The consumption of water or lipid extracts of oleander in folk medicine practices have contributed to considerable incidences of intoxication (Hamouda et al., 2000; Wojtyna and Enseleit, 2004; Tor et al., 2005; Wasfi et al., 2008; Kozikowski et al., 2009; Al et al., 2010; Kolkhof et al., 2010; Renier et al., 2013). Adults are fatally poisoned upon consuming 5–15 *N. oleander* leaves or 3 g of dried leaves (Pietsch et al., 2005; Bandara et al., 2010; Liu, 2016), and one leaf may be lethal for children (Shaw and Pearn, 1979; Wasfi et al., 2008). Likewise, most oleander poisoning incidents in livestock are caused by the accidental ingestion of oleander leaves. The ingestion of oleandrin or oleander produces typical clinical symptoms of CG poisoning (Wasfi et al., 2008; Roberts et al., 2016), and inhibition of cardiac Na/K-ATPase is the main mechanism of toxicity (Schoner and Scheiner-Bobis, 2007; Abdou et al., 2019). The toxic blood concentration of oleandrin for humans is estimated to be between 1 and 2 ng/ml (Pietsch et al., 2005), and the fatal blood concentration is approximately 20 ng/ml (Wasfi et al., 2008).

The clinical application of oleandrin is also limited by its low water-solubility and significant toxic side effects. However, greater attention has been drawn to the novel antiviral effects of oleandrin because of the recent coronavirus disease (COVID-19) pandemic caused by SARS-CoV-2 worldwide. There is currently a lack of systematic pharmacokinetic reports of oleandrin. Additional studies on the pharmacokinetic profile of oleandrin is necessary for the development of safe clinical applications. To date, only poisoning cases and sporadic animal experiments provide a basis for an in-depth understanding of the pharmacokinetic properties of oleandrin *in vivo*. The physicochemical properties, formulations of oleandrin, individual genetic polymorphisms, and the composition of gut microbiota appear to have significant effects on the pharmacokinetic behavior of oleandrin (Zhou et al., 2019; Dey, 2020). Through modern preparation techniques and personalized medicine, understanding the pharmacokinetic behavior of oleandrin may provide effective strategies for solving the pharmacological issues currently associated with oleandrin.

All available information on oleander or oleandrin was collected from Science Direct, China National Knowledge Infrastructure (CNKI), PubMed, Google Scholar, Baidu Scholar, the Web of Science, and related books. In this review, we summarized the sources, structural features, detection methods, pharmacokinetics (absorption, distribution, metabolism, and excretion [ADME]), and toxicology of oleandrin in order to provide a theoretical basis for further research and development of the glycoside.

## 2 Natural Sources and Structural Properties of Oleandrin

**FIGURE 1:**
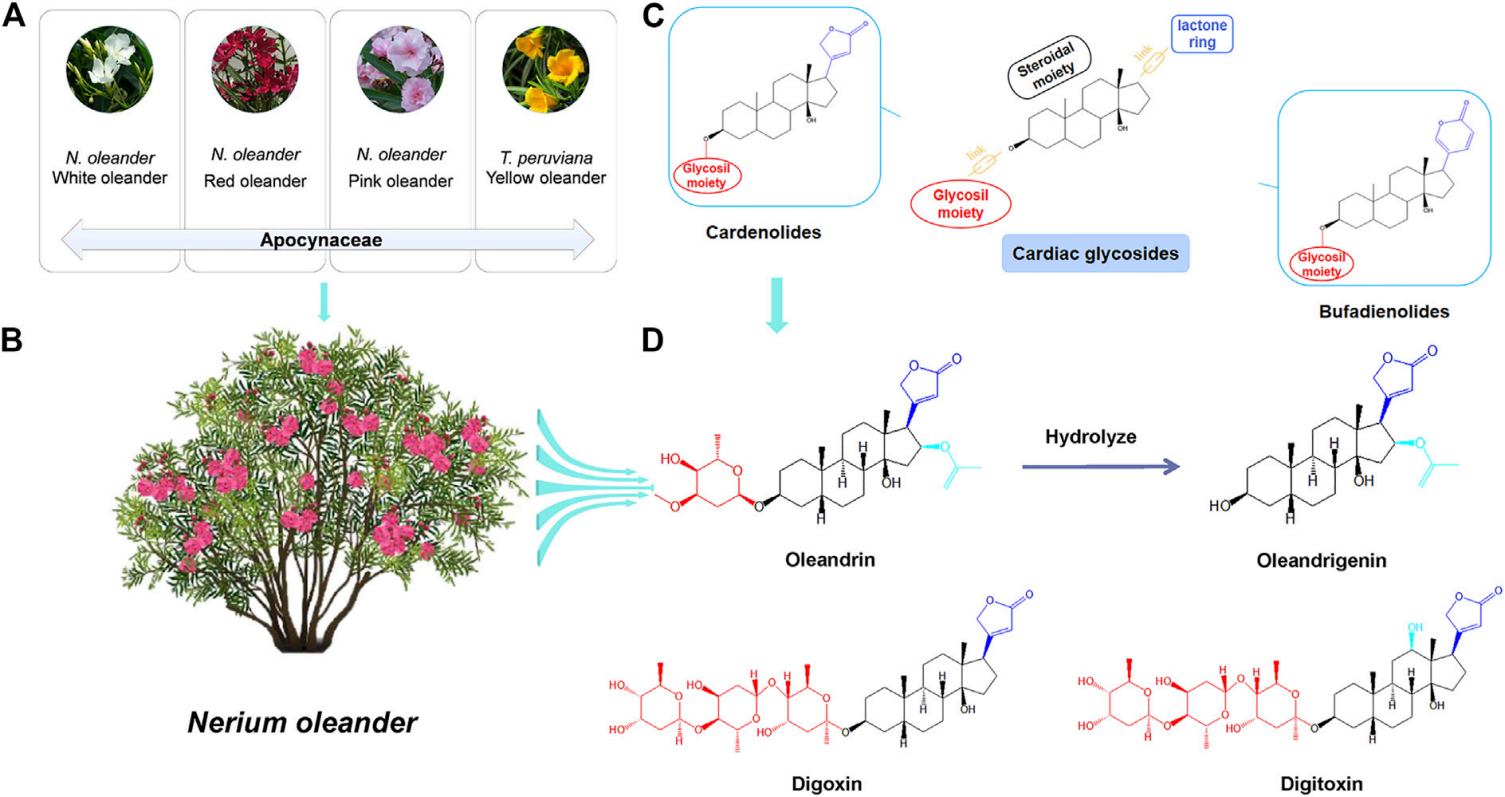
Natural sources and structural properties of oleandrin. **(A)** Categories of plants commonly known as “oleander.” **(B)** The main sources of oleandrin: *Nerium oleander*. **(C)** Classification of cardiac glycosides (CGs). **(D)** The chemical structure of oleandrin and its hydrolysate oleandrigenin and other structure of typical CGs.

Oleandrin,16-(acetyloxy)-3-[(2,6-dideoxy-3-O-methylhexopyranosyl)oxy]-14-hydroxycard-20(22)-enolide (CAS Registry No.559-83-1), was the first CG and main active component isolated from *N. oleander* by Lukowski in 1861 (Fang, 2013). *N. oleander* plants mainly have red, white, or pink flowers (Figure 1A) (Khan et al., 2010). Red-flowered oleander (Figure 1B) varieties exhibit higher levels of CGs than those of white-flowered varieties (Karawya et al., 1973; Langford and Boor, 1996). Oleandrin is present in all parts of the *N. oleander* plant, including the stems, leaves, flowers, buds, nectar, and sap (Karawya et al., 1973; Langford and Boor, 1996). Heat does not inactivate the glycoside; therefore, oleandrin is even found in burned products of *N. oleander* (Langford and Boor, 1996; Khan et al., 2010). The concentration of oleandrin varies according to the part of the plant, and the highest concentration is found in the leaves (Langford and Boor, 1996). *Thevetia peruviana* is known as yellow oleander (Figure 1A), but is a separate species belonging to the same family, Apocynaceae, and does not produce oleandrin (Langford and Boor, 1996). Thevetin A is the main active component of yellow oleander (Langford and Boor, 1996).

Oleandrin (C_32_H_48_O_9_) is a white crystalline powder with a melting point of 250°C and a molecular weight of 576.727 Da, and it is insoluble in water and soluble in methanol, ethanol, and chloroform. Oleandrin, digoxin, and digitoxin are typical CGs; their structural formulas are shown in Figure 1D. Oleandrigenin is a deglycosylated metabolite of oleandrin (Ni et al., 2002). CGs are naturally derived compounds with broad pharmacological activities that show considerable structural diversity. However, they have a common structural motif and generally consist of three structural features: An aglycone-steroidal moiety, a lactone ring with five or six carbons, and a glycosil moiety (McConkey et al., 2000; Ruta et al., 2020). The aglycone-steroidal moiety is considered the pharmacophoric moiety responsible for the activity of CGs (Schonfeld et al., 1985). The difference in the lactone ring at C-17 defines the class of CGs. Generally, CGs are divided into cardenolides and bufodienolides (Figure 1C) (Prassas and Diamandis, 2008). Cardenolides contain a five-membered unsaturated butyrolactone ring and include oleandrin, digoxin, and digitoxin, whereas bufodienolides contain a six-membered unsaturated pyrone ring as observed in bufalin. CGs also possess rare glycosil moieties, such as rhamnose and digitalose (Prassas and Diamandis, 2008). Slight structural differences play an important role in variations in the toxicokinetics and toxicodynamics of these substances (Group, 1997). For example, free aglycones are absorbed faster and are easier to metabolize than their glycosylated counterparts (Prassas and Diamandis, 2008). Oleandrin contains a central steroid nucleus with an unsaturated lactone structure on the C-17 atom and a dideoxy arabinose group at C-3. However, the three sugars at C-3 of digoxin and digitoxin are digitoxose (Figure 1D) (Jortani et al., 1996; Jia et al., 2014). Different types of sugars at C-3 affects the pharmacodynamic and pharmacokinetic profile of CGs (Prassas and Diamandis, 2008). The configuration of the important substituents at C-3, C-14, and C-17 of the CG structures has a significant impact on their pharmacological activity (Prassas and Diamandis, 2008). For example, digitoxin is less polar than digoxin because of the lack of a hydroxyl group (Jia et al., 2014). Upon analyzing the molecular structure of oleandrin, we found that its polarity was also lower and its lipid solubility was higher than that of digoxin. It is speculated that the pharmacokinetic behavior of oleandrin is closer to that of digitoxin than digoxin.

## 3 Detection Methods

Highly sensitive and reliable detection methods for trace-level oleandrin and its metabolites in biological samples are the basis of pharmacokinetic studies. The sensitivity of oleandrin detection technology has increased with time (shown in Table 1). These methods include thin-layer chromatography (Blum and Rieders, 1987; Galey et al., 1996; Holstege et al., 2000), digoxin immunoassays (Dasgupta and Datta, 2004), fluorescence spectrophotometry (Dasgupta and Hart, 1997), high-performance liquid chromatography (Hamad et al., 2002), and liquid chromatography mass spectrometry (LC-MS) (Tracqui A, 1998; Kanno et al., 2011). Liquid chromatography-tandem mass spectrometry (LC-MS/MS) with multiple reaction monitoring (MRM) is the preferred detection method for the analysis of complex and difficult-to-volatile CGs in biological samples (Wang et al., 2000; Tor et al., 2005; Josephs et al., 2010; Kanno et al., 2011; Ying et al., 2017; Zhai et al., 2018; Gosetti et al., 2019).

**TABLE 1:** Summary of oleandrin detection methods.

Compounds	Samples	Species	Column	Pretreatment	Detection method	LOD(ng/mL or ng/g)	Recovery	Ref
Oleandrin	Serum	Bovine	Una C_18_ column	LLE	LC-MS/MS	1	97 ± 5%	Tor et al. (2005)
	Urine			SPE			107 ± 7%	
	Liver						98 ± 6%	
Oleandrin	Plasma	Human	WatersNovaPak 4-µmC_18_ column	SPE	LC-MS/MS	1	90%[ouabain (IS)]	Wang et al. (2000)
Oleandrin Desacetyloleandrin Oleandrigenin Gitoxigenin	Blood	Human	GH-Cls(III) column	LLE	LC-3DQMS	3	76.7 ± 3.1%	Arao, (2002)
					LC-MS/MS	2	87.6 ± 1.2%	
						2	82.3 ± 3.6%	
						30	21.2 ± 1.8%	
Oleandrin et al. 18 plant toxins	Beverages	Beverages	Mastro C_18_ column	QuEChERS	LC-MS/MS	N/A	74–108%	Oishi et al. (2019)
Oleandrin	Blood	Human	Reversed-phase Hypersil Gold C_18_ column	LLE	LC-MS/MS	1	>90%	Wasfi et al. (2008)
	Oleander leaves							
Oleandrin	Urine	Livestock	N/A	SPE	2D-TLC	20	N/A	Ceci et al. (2020)
	Ingesta					50		
Oleandrin	Serum	Human	Synergy4μ Polar-RP 80A column	SPE	LC-MS/MS	1(LOQ)	62 ± 6%	Pietsch et al. (2005)
	Urine					1.2(LOQ)		
Oleandrin	Blood and tissues	Human	N/A	LLE	TLC	N/A	N/A	Blum and Rieders, (1987)
					Fluorescence			
					Spectrophotometry			
Oleandrin	Oleander leaf, serum	Human	N/A	LLE	Digoxin immunoassays	N/A	N/A	Dasgupta and Datta, (2004)
Oleandrin	Blood	Human	Waters NovaPak C_18_ column	LLE	LC-MS	0.4(LOQ)	86.8 ± 8.3%	Tracqui A, (1998)
	Urine							
Oleandrin	Blood	Human	Agilent Zorbax SB-C_18_ column	LLE	LC-MS/MS	1	>70.50%	Zhai et al. (2018)
	Liver					2		
Oleandrin and other CGs	Herbs	Human	AQUITY UPLCTM BEH C_18_ column	SPE	LC-MS/MS	1.5(LOQ)	70–120%	Malysheva et al. (2020)
	Urine					0.025(LOQ)		
Oleandrin and other CGs	Blood	Human	Zorbax DB-C_18_ column	LLE	LC-MS/MS	1	88%	Carfora et al. (2021)
	Urine							
Oleandrin	Milk	Cows	Acquity UPLC BEH Shield column	SPE	UPLC-MS/MS	0.018 0.010	74.8 ± 5.8%	Ceci et al. (2020)
	Cheese						70.5 ± 4.9%	
Oleandrin	Blood, serum, heart and liver	Bovine	Acquity UPLC BEH Shield column	SPE	UHPLC-MS/MS	0.11	62.9–80.5%	Gosetti et al. (2019)
Oleandrin Adynerin	Blood	Human	Agilent, Eclipse Plus C_18_ column	Protein Precipitation	LC-MS/MS	0.5	75.2–95.7%	Song et al. (2017)
Oleandrin Adynerin	Blood	Human	Kinetex C_18_ column	Solid phase supported LLE	LC-MS/MS	0.5	90.0–98.0%	Ying et al. (2017)
Oleandrin	Blood	Bovine	Shimadzu STR ODS II column	SPE	LC	1.5	>88.8%	Hamad et al. (2002)
Oleandrin and eight alkaloids	Herbal cosmetics	Herbal cosmetics	Waters UPLC	SPE	LC-MS/MS	1.0(LOQ)	86.9–116.5%	Xun et al. (2017)
			HSS T3 column					
Oleandrin	Heart, liver, kidneys and brain	Caviaporcellus	Shimadzu Shimpack XR-ODSII column	QuEChERS	UFLC-MS/MS	1	N/A	Botelho et al. (2018)
Oleandrin	Blood	Human	Zorbax SB C_18_ column	LLE	LC-MS/MS	N/A	N/A	Azzalini et al. (2019)

LC-MS/MS combines the advantages of the high separation efficiency of LC and the strong compound identification capabilities of MS. Detection efficiency has been improved by a combination of electrospray ionization with MS, owing to the gentle ionization under atmospheric pressure conditions. CGs may be analyzed even in complex human matrices in MRM mode (Dey, 2020). Owing to the sensitivity and specificity of LC-MS technology, attempts were made to use this method in as early as 1993 to confirm oleandrin in decayed human tissues (Rule et al., 1993). Recent studies have indicated that trace-level oleandrin can be rapidly and accurately analyzed in various complex biological samples, both qualitatively and quantitatively using LC-MS/MS (Song et al., 2017; Xun et al., 2017; Ying et al., 2017; Botelho et al., 2018; Zhai et al., 2018; Gosetti et al., 2019; Oishi et al., 2019; Ceci et al., 2020). As such, LC-MS/MS analysis has evolved into the best choice for pharmacokinetic and experimental toxicological studies of oleandrin (Tor et al., 2005). Oleandrin concentrations have been assessed in various biological samples, including the heart, bile, peripheral blood, urine, liver, and gastric content, where the LC-MS/MS detection limit reached 0.01 ng/ml. In addition, liquid-liquid extraction or solid-phase extraction was the most common pretreatment method, exhibiting the advantages of simplicity and ideal extraction recovery rate (Tor et al., 2005; Wasfi et al., 2008; Zhai et al., 2018; Malysheva et al., 2020; Carfora et al., 2021). These factors make this sensitive and specific method a powerful tool for toxicology and toxicokinetics studies in humans and livestock, and may provide key information to clinicians and veterinary practitioners regarding oleandrin toxicity. However, oleandrin is currently the only constituent isolated from oleander that is available as a pure standard (Tor et al., 2005). Other toxic components of oleander, such as neritaloside, oleandrigenin, and odoroside standards are not available commercially, and only LC/QqTOF or Orbitrap MS have been applied for their preliminary qualitative assessments in some studies (Wang et al., 2000; Carfora et al., 2021). The identification of all the metabolites of oleandrin and other toxic components of oleander is also of great significance for the study of oleander pharmacology.

## 4 Pharmacokinetics

Pharmacokinetics is mainly composed of two interconnected parts. The first is the drug disposition by the body, that is, ADME of drugs *in vivo* over time; the second is the principle of drug application kinetics and mathematical models that quantitatively describe the law of blood drug concentration changes over time and the rate of drug treatment in the body (Zhu and Yin, 2016). Systems pharmacokinetics is an interdisciplinary field of study that creates synergy at the interface between systems biology and pharmacokinetics. Systems pharmacokinetics descripts models and deals with discovering emergent properties of enzymes, cells, tissues, and the body as an integral system (Gao et al., 2014). Starting from the time of initial blood concentration of a drug after a single dose, the relationship between absorption, distribution, metabolism, excretion, and blood drug concentration can be determined. Pharmacokinetic studies can aid in the precise calculation of dosing regimens, such as the dosage and dosing interval. Kanwal in 2020 pointed out that *in vivo* and in silico studies are required to explore the mechanistic approaches regarding the pharmacokinetics and biosafety profiling of “botanical” drugs to completely track their candidature status in drug discovery with natural substances (Kanwal et al., 2020).

**FIGURE 2:**
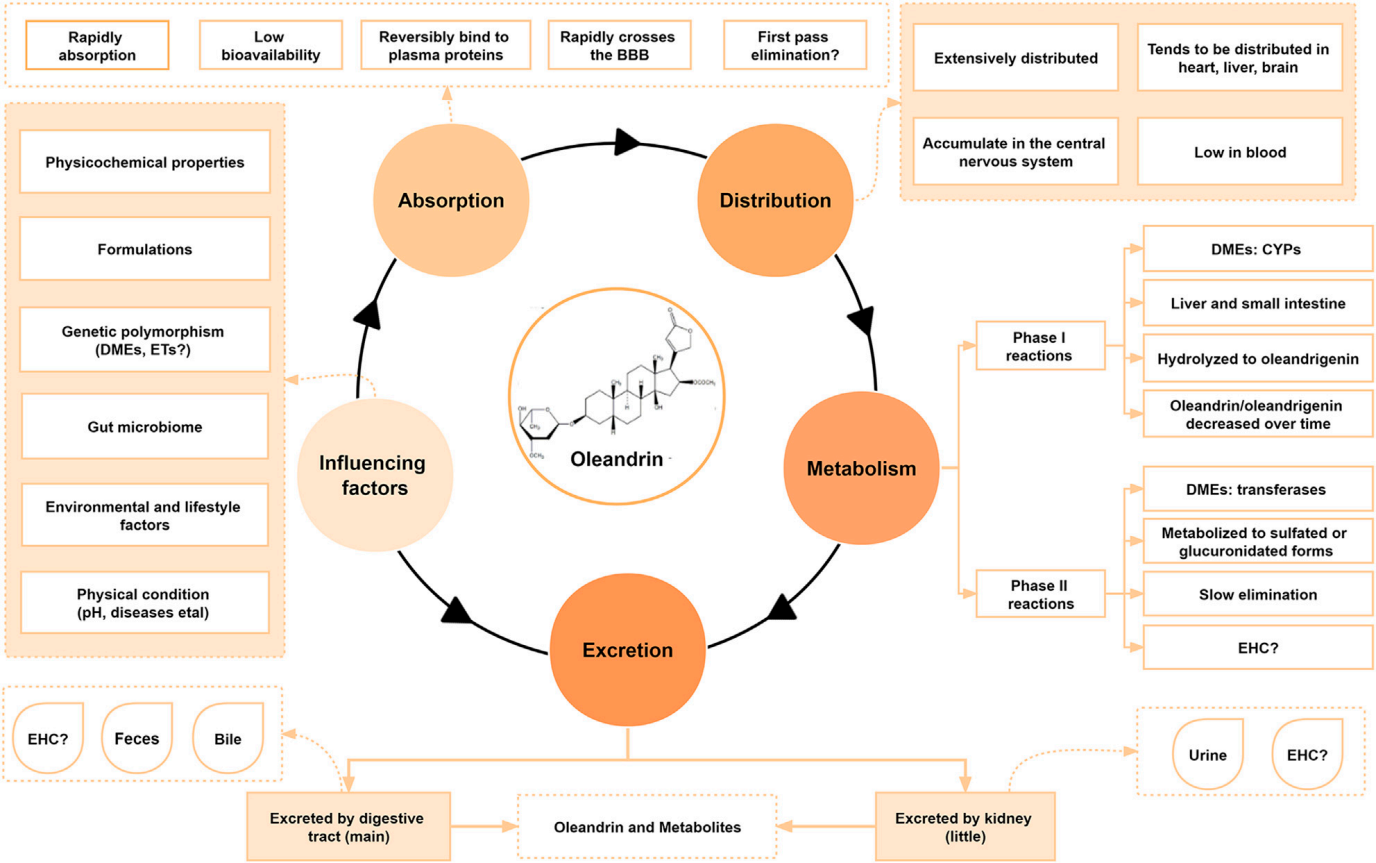
Pharmacokinetic characteristics and influencing factors of oleandrin.

**FIGURE 3:**
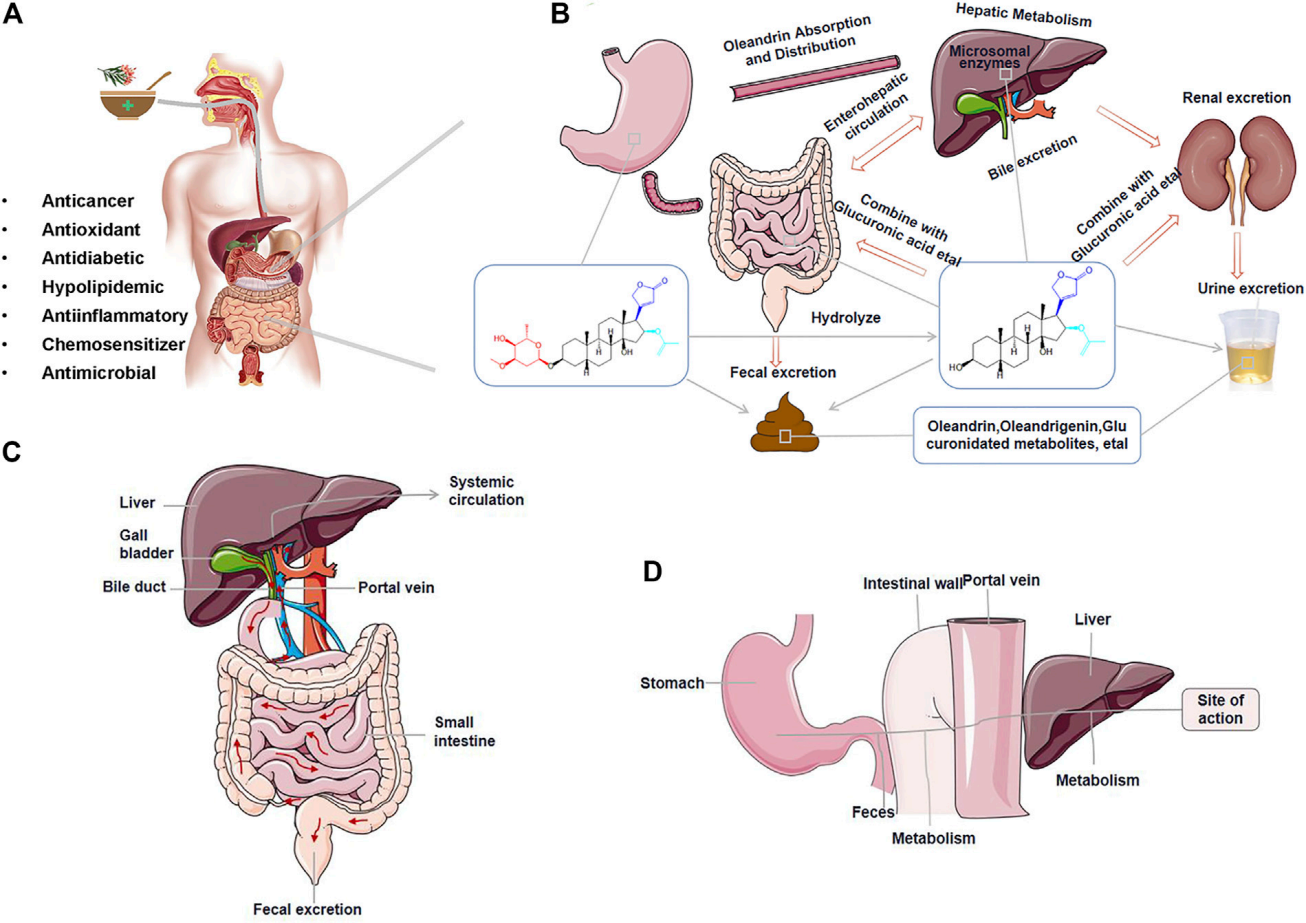
The absorption, distribution, metabolism, and excretion (ADME) process of oleandrin. **(A)** Pharmacological properties of oleandrin. **(B)** Detailed ADME process of oleandrin *in vivo*. **(C)** The possibility of bile excretion and enterohepatic circulation (EHC) of oleandrin *in vivo*. **(D)** The possible first-pass effect.

The pharmacokinetics of CGs have been studied in rabbits, rats, and humans (Hinderling and Hartmann, 1991; Ni et al., 2002; Jia et al., 2014; Garcia-Iranzo et al., 2017). However, little is known about the pharmacokinetic behavior of oleandrin in animals, especially humans, since only a few poisoning cases and sporadic animal experiments have been analyzed. To date, the reported pharmacokinetic characteristics of some CGs are closely related to their physicochemical properties, formulations, individual gene polymorphism such as the expression of P-glycoprotein (P-gp) (Caloni et al., 2012; Zhou et al., 2019), and the composition of the gut microbiota (Zhang et al., 2018; Dey, 2020). The pharmacokinetic characteristics and influencing factors of oleandrin collected by various studies are summarized in Figures 2, 3B.

### 4.1 Absorption

Absorption refers to the process by which a drug enters the blood circulation from the site of administration. The rate and degree of drug absorption depend on the physicochemical properties of the drug, formulation, dose, and route of administration. Oral drugs are typically absorbed by the gastrointestinal mucosa (Zhu and Yin, 2016). A drug is absorbed through the capillary wall after subcutaneous or intramuscular injection. Most drugs can readily pass through the capillary wall because of the large intercellular space, allowing for quick and complete absorption. Drugs have three potential membrane transport modes: Passive diffusion, facilitated diffusion, and active transport. Simple diffusion, a type of passive diffusion, is the primary membrane transport method for most low-molecular-weight drugs, and it is the usual mode of drug uptake in the small intestine and stomach (Patocka et al., 2020). The more fat-soluble the drug, the faster the diffusion rate (Zhu and Yin, 2016). The first-pass effect caused by high drug permeability in the intestine and saturation of intestinal absorption mediated by transporters could drive low oral bioavailability of the drug (Figure 3D) (Thompson et al., 2019).

Conventionally, oleander is administered orally as a decoction to treat various diseases in humans and animals (Figure 3A) (Songu-Mize et al., 1983; Hauck et al., 2009). Oleandrin acts in a time-dependent and dose-dependent manner (Kucukdurmaz et al., 2012; Roberts et al., 2016; Botelho et al., 2020). After oral administration, oleandrin is first absorbed in the oral mucosa by simple diffusion, and then quickly absorbed in the gastrointestinal tract, resulting in immediate effect (Liu, 2016). Ni in 2002 showed that oleandrin was rapidly absorbed after oral administration (maximum serum concentration [C_max_] at 20 min) with an oral bioavailability of approximately 30% (Ni et al., 2002). They also found that the C_max_ in mice was reached in <0.5 h after an intraperitoneal (i.p.) dose of oleander extract, whereas humans reached the C_max_ within 3 h after an intramuscular dose of oleander extract (Ni et al., 2002). However, orally administered oleandrin has a longer elimination half-life than its intravenous (i.v.) dose. As such, it was hypothesized that these differences in absorption may be related to the species or the ingestion routes (Ni et al., 2002). A 2009 study reported that oleandrin had a better absorption rate in species with higher expression levels of the Na/K-ATPase α3 subunit protein (Yang et al., 2009; Colapietro et al., 2020).

The half-life of oleandrin in humans is 2.3 h (Ni et al., 2002). The half-life of digoxin *in vivo* varies from species to species (Group, 1997); which is speculated to be the case for oleandrin too. The lipophilic properties of oleandrin allow it to easily pass through the blood–brain barrier (BBB). The lipophilic property was demonstrated by showing the presence of oleandrin in brain tissues as early as 30 min after i.p. injection (Ni et al., 2002). The length and structure of the small intestine, with its villi and microvilli, makes it more effective than the stomach in absorbing compounds. Therefore, the absorption of drugs, such as CGs, primarily occurs in the small intestine. Oleandrin may be efficiently transported across the apical membrane of intestinal epithelial cells via active transport.

The lipophilic properties of oleandrin allow it to be easily accumulated and eliminated slowly (Songu-Mize et al., 1983). A variety of highly fat-soluble CGs undergo enterohepatic circulation (EHC) (Figure 3C) (Jia et al., 2014). EHC is a process through which some drugs eliminated by hepatocytes enter the intestine via the bile, are reabsorbed from the intestine into the portal vein, and then return to the liver to enter the systemic circulation (Gao et al., 2014). EHC can prolong the elimination half-life of glycosides leading to accumulation and poisoning (Jia et al., 2014). It has been speculated from limited studies that oleandrin also exhibits EHC, where oleandrin may be reabsorbed into the portal vein and enter the systemic circulation in the terminal ileum.

The low bioavailability of oleandrin may be due to its poor water solubility, its reversible and rapid binding to plasma proteins, or possible P-gp-mediated efflux and first-pass effect (Figure 3D) (Jia et al., 2014). P-gp, a widely expressed glycoprotein in epithelial cells or normal tissues, is involved in the disposition of oleandrin. Zhou in 2019 revealed that the key factor determining the toxicity level of oleandrin is the difference in the expression of intestinal P-gp over time. The toxicity level of oleandrin can be minimized by optimizing the administration time (Zhou et al., 2019). New technologies or formulations can be used to increase oleandrin oral bioavailability by decreasing the first-pass effect to enable higher oral oleandrin doses and by reducing the saturability of oleandrin intestinal absorption. These technologies include the combined application of absorption enhancers and formulating oleandrin into solid lipid nanoparticles or transporter-targeted pro-drugs. Sustained-release preparations would theoretically extend the absorption period of a drug and thus increase the accumulated uptake, thereby increasing overall exposure. The polarity of oleandrin can be changed by modifying its structure. The route of administration of the glycoside can also be changed by sublingual administration or injection of oleandrin in divided doses to avoid the first pass effect, thereby improving bioavailability. Ideal bioavailability and therapeutic effects can also be achieved through personalized dosing regimens. Further research needs to be done to understand the mechanistic basis of oleandrin’s low oral bioavailability.

### 4.2 Distribution

When a drug enters the bloodstream, the process of permeating from the blood capillaries to the tissue begins immediately and is called drug distribution (Gao et al., 2014). Distribution of drugs via body circulation to various tissues, organs, or body fluids is selective, unevenly distributed in various tissues and organs, and has a dynamic balance (Gao et al., 2014). The apparent volume of distribution reflects the degree of localized distribution of the drug. A variety of non-polar CGs with high fat-solubility exhibit EHC during the absorption process. For example, digitoxin is easily absorbed from the gastrointestinal tract and undergoes EHC. Since the binding of digitoxin to plasma proteins is weak, it is widely and rapidly distributed in various tissues in the form of metabolites, especially in the liver, gallbladder, and intestines. This phenomenon is closely related to EHC (Jia et al., 2014). However, digoxin is highly water-soluble, not easily absorbed through the gastrointestinal tract, and rarely undergoes EHC. Hence, digoxin is rarely distributed in the brain, owing to its low fat solubility and difficulty in passing through the BBB (Jia et al., 2014). This confirms that the chemical structural characteristics of a drug play an important role in its pharmacokinetic behavior. There has been a global agreement on the need to perform a single-dose tissue distribution study as part of preclinical programs to provide information about tissue distribution of the investigational drugs (Gao et al., 2014).

There is a lack of systematic reports on the tissue distribution of oleandrin and its metabolites since its distribution characteristics have been mostly studied in poisoning cases and animal experiments. Studies in rats revealed that oleandrin enters the blood, reversibly binds to plasma proteins, and is then distributed throughout the body (Jia et al., 2014). Ni in 2002 showed that oleandrin can rapidly accumulate in the central nervous system in mice, passing through the BBB (Ni et al., 2002). Even after a single injection, the observed concentration of oleandrin in the brain tissue is very high, and it continues to increase over time within 24 h (Ni et al., 2002). Therefore, the symptom of severe vomiting caused by oleandrin poisoning may also be due to the penetration of the BBB and stimulation of the medulla oblongata chemosensory area. The concentration of oleandrin in the brain was found to be higher than that of the original oleander extract after administering the same dose of oleander extract and oleandrin (Ni et al., 2002). It was speculated that components within the oleander extract may have enhanced the transport of oleandrin across the BBB. Regarding the presence of oleandrin in milk, Ceci in 2020 showed that the glycoside was detected in milk and cheese from dairy cattle. This demonstrated that oleandrin can also across the blood–milk barrier (Ceci et al., 2020). Ni in 2002 also demonstrated that the concentration of oleandrin in the liver was approximately twice of that in heart or kidney tissues (Ni et al., 2002). However, Liu in 2016 reported that oleandrin was mostly distributed in the heart and skeletal muscle after absorption (Liu, 2016), perhaps explaining its potential cardiotoxicity (Songu-Mize et al., 1983; Ada et al., 2001; Hauck et al., 2009).

**FIGURE 4:**
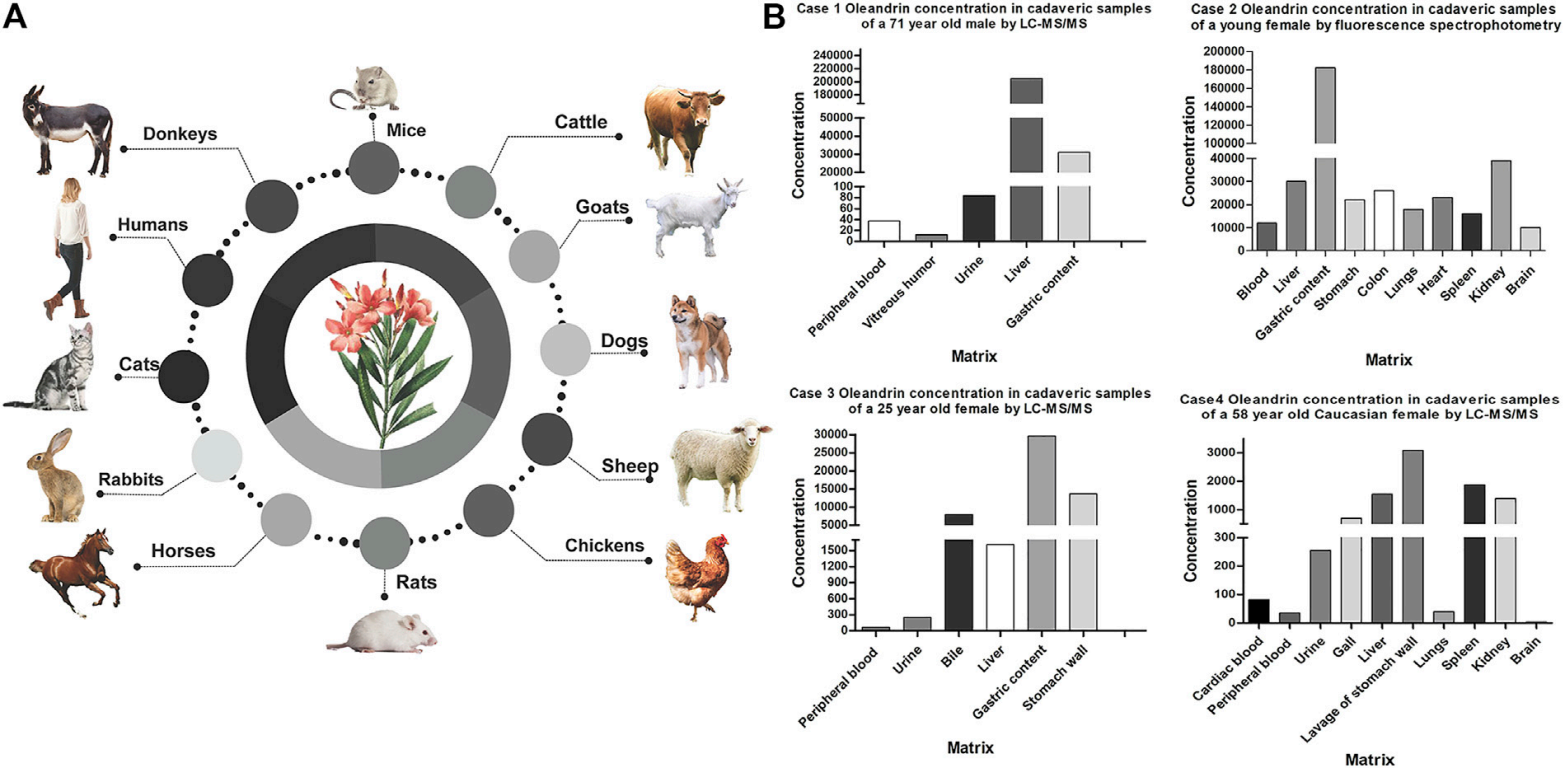
Overview of literature data of the poisoning cases caused by Nerium Oleander. **(A)** Species involved in oleander poisoning cases. **(B)** Summary of oleandrin tissue distribution concentration in reported fatal cases of oleander in humans (ng/mL or ng/g).

Some fatal cases of oleandrin intoxication have been reported (shown in Figure 4). The differences in oleandrin concentration detected in similar samples obtained in various cases, or in different samples, may be closely related to the postmortem process and the time of death (Sosabowski, 2004). The results of reported cases indicated that oleandrin targeted the heart, liver, and brain (shown in Figure 4B); however, there was large inter-individual variability (Carfora et al., 2021; Blum and Rieders, 1987; Zhai et al., 2018; Azzalini et al., 2019). The detection results of several biological samples case 3 of Figure 4B showed that the concentration of oleandrin was the highest in the gastric content, followed by stomach wall, bile, liver, and urine; the lowest concentrations were found in the heart and blood (Zhai et al., 2018). The concentration of oleandrin in the gastric content and stomach wall becomes very high on oral administration. The low proportion of oleandrin absorbed into the blood indicates its low bioavailability (Plante et al., 2020). The high concentration of oleandrin in the bile may be related to the rapid excretion of oleandrin through the biliary tract after intragastric administration. Published data have also shown that oleandrin easily accumulates in the body and elimination occurs mainly through the feces (Ni et al., 2002). From this, we can infer that the metabolism of oleandrin may also involves EHC (Soto-Blanco et al., 2006), leading to a long half-life, accumulation, and subsequent poisoning. As such, bile is an effective specimen for the identification of oleandrin in cases of oleander poisoning. Although apart of the drug is excreted by the kidneys during EHC, low levels of oleandrin in the urine indicate low kidney organ exposure.

In addition to oleandrin, some of its metabolites such as oleandrigenin also have dynamic distribution characteristics in the body. Animal experiments by Blum in 1987 showed that the active metabolites were mostly concentrated in the liver after i.v. injection of oleandrin in mice (Blum and Rieders, 1987). Ni in 2002 showed that the content of oleandrin in the liver was >60% after 5 min of administration in mice, while the content of oleandrigenin was 28%. Moreover, oleandrin and oleandrigenin exist in both urine and feces, with the latter having a higher concentration of the compounds. As such, the liver is more exposed to oleandrin because the main route of excretion occurs via bile into the feces, while the kidneys are less exposed because oleandrin urine excretion is a minor pathway (Songu-Mize et al., 1983).

### 4.3 Metabolism

The metabolic process of drugs is usually divided into two phases: Phase I and phase II biotransformation (Zhu and Yin, 2016). Phase I biotransformation reactions are primarily functional group reactions, including oxidative metabolism (oxidation of aromatic rings, olefins/alkynes, alkyl groups, amino groups, ethers, and thioethers), reduction reactions, and hydrolysis reactions. In phase II biotransformation, some polar groups of the pro-drug or metabolites after functionalization are covalently bonded with endogenous small molecules in the presence of enzymes, producing highly polar conjugates that are easily excreted from the body. The combination reaction converts the drug to water-soluble metabolites, which are beneficial for excretion via urine or bile or for deactivating the metabolites. The combination reaction is an important process for drug inactivation or elimination (You, 2016). After the process of drug absorption and circulation, structural transformation via metabolism occurs as a result of the activity of intestinal flora or enzymes. Drugs with high fat-soluble properties are metabolized to produce low fat-soluble or high water-soluble metabolites which are easily excreted from the body. Enzymes involved in drug metabolism include microsomal enzymes and non-microsomal enzymes in different locations. The biotransformation of CGs is primarily carried out under the action of microsomal enzymes in the liver (Group, 1997). Typically, some CGs undergo a hydrolysis reaction in phase I of metabolism, while others undergo hydroxylation and lactone ring hydrogenation reactions. The metabolites produced by hydrolysis and hydroxylation are still active, whereas the metabolites obtained by hydrogenation have weak or no cardiotonic effects. The phase I metabolites generated in the presence of phase II enzymes then react with endogenous small molecules, such as glucuronic acid or sulfuric acid, to form phase II metabolites, which are excreted from the body (Jia et al., 2014). However, if the CG undergoes EHC, its glucuronide conjugates are cleaved by microbiome-encoded β-glucuronidases, resulting in reabsorption (Tsunoda et al., 2021).

Pharmacokinetic studies of the representative CG digoxin are relatively advanced. After oral administration, digoxin is absorbed in the upper part of the small intestine and is excreted from the kidneys in its original form. Approximately 7% of the total absorbed concentration is metabolized by the liver, producing hydrogenated dihydrodigoxin, which is then hydrogenated to dihydrodigoxigenin, and hydrolyzed into different products. Finally, metabolites are combined with glucuronic acid and excreted by the kidneys (You, 2016). The biotransformation of oleandrin occurs in the liver and small intestine, where it is metabolized by liver microsomal enzymes (Liu, 2016). The C-17 position of oleandrin, digoxin, and digitoxin are all five-membered unsaturated lactone rings that are easily hydrolyzed into aglycones and free sugars under mild acidic conditions. Oleandrigenin, an aglycone of oleandrin, is the main metabolite (Ni et al., 2002). Ni in 2002 speculated that the metabolism of oleandrin to oleandrigenin is an enzymatic process (Ni et al., 2002), possibly a hydrolysis reaction catalyzed by hepatic or intestinal enzymes. Oleandrin is linked to arabinose, and cytoplasmic-glucosidase, which is found in the intestine, can hydrolyze arabinose *in vitro* (Berrin et al., 2002). Cytoplasmic-glucosidase may play a role in promoting the hydrolysis of oleandrin-glucuronide conjugates, resulting in EHC (Ashford, 2002). After a single dose of oleandrin is administered, monitoring the changes in blood concentration over time. If oleandrin undergoing EHC, it will show the multiple-peak phenomenon in its blood-concentration–time profile and the prolonged elimination half-life (Gao et al., 2014). In addition, intestinal disposition may affect the hepatic disposition in the first-pass metabolism of oleandrin. The gastrointestinal tract influences the metabolism and conjugation of oleandrin before its entry into systemic circulation and the liver.

Compounds containing hydroxyl, amino, carboxyl, heterocyclic nitrogen, and sulfhydryl in phase II metabolism can be combined with endogenous small molecules, such as glucuronic acid, sulfuric acid, amino acids, and glutathione. Glucuronic acid is easily soluble in water, and compounds with polar functional groups can be combined with glucuronic acid. The combined product is extremely water soluble and is typically excreted through the urine. However, when the molecular weight of the combined product is greater than 300 Da, it is excreted through the bile (Watson et al., 2003). The combined chemical structure of oleandrin contains a hydroxyl group, and its molecular weight is 576 Da. The threshold molecular weight of drugs undergoing the biliary route of excretion is estimated to be 500–600 Da (Roberts et al., 2002). Enterohepatic recycling can often be accompanied by hepatic conjugation and intestinal deconjugation (Tsunoda et al., 2021). Oleandrin is first partially hydrolyzed to oleandrigenin, improving its absorption, and then extensively metabolized by enzymes producing glucuronated or sulfated forms, before reaching the systemic circulation (Ni et al., 2002; Hostetler et al., 2017). Conjugation reactions may occur in the liver and intestine, and oleandrin and its metabolites can then be transported to the basolateral side of the small intestine. The oleandrin conjugate may be de-conjugated by enzymes encoded by the gut microbiota and the products transported to the liver through the hepatic portal vein, initiating EHC (Gao et al., 2014; Wang et al., 2021). Oleandrin can be further hydrolyzed and metabolized by bacteria in the colon. The concentration of oleandrigenin gradually increases, while the corresponding concentration of oleandrin gradually decreases over time (Ni et al., 2002). A study reported that by 4 h after i.v. injection of oleandrin, oleandrigenin could still be detected in mouse heart and kidney tissues, but oleandrin could not be detected (Ni et al., 2002). This indicates that the metabolic half-life of oleandrin in the body is shorter than that of its metabolite oleandrigenin. Since the proportion of metabolites excreted is relatively large, the study of oleandrin metabolites is of great significance. It is worth noting that no metabolites, including oleandrigenin, can be detected in the brain tissue (You, 2016). This is expected since only high-fat-soluble, non-dissociated substances can easily pass through the BBB (Ni et al., 2002).

As indicated, the elimination rate of oleandrin is relatively slow, and oleandrin maintains its activity in the body for a long time, potentially resulting in accumulation poisoning. A study had measured the blood concentration-time profile of oleandrin and relative concentration-time profiles of neritaloside, oleandrigenin, and odoroside in a volunteer. After the volunteer took 15 mg of oleandrin, the blood concentration reached the highest peak of 7 ng/ml at 3 h. This concentration of oleandrin remained virtually asymptotic for the time trial of the study, thereby suggesting that there is a slow clearance of this CG from human plasma (Wang et al., 2000). In addition, oleandrin is highly bound to plasma proteins, which also prolongs the elimination half-life. After the drug is bound to the plasma protein, the large complex experiences difficulty passing through the capillaries, so it is temporarily stored in the blood. This results in slow transport (distribution or excretion), which prolongs the time of action of the drug. However, this conjugation is reversible, and there is a dynamic balance between the bound and free drug in the plasma. When this balance is disrupted, concentration of the free drug in the blood increases, which can lead to toxic reactions (Fang, 2013). The above studies infer that oleandrin will undergo EHC, which results in a bimodal or multimodal blood concentration-time curve, thereby prolonging the elimination of oleandrin.

Many factors affect drug metabolism. The gut microbiota directly and indirectly participate in pharmacokinetics of drugs. For example, bile acids can be modified by bacteria in the gut and influence regulation of host metabolism. Hinderling in 1991 indicated that the formation of digoxin metabolites is related to the effects of the intestinal flora. Many digoxin metabolites can be detected in healthy subjects after p.o. administration, whereas fewer metabolites are produced after i.v. administration (Hinderling and Hartmann, 1991). The route of administration has a significant impact on the metabolic process of digoxin, but oleandrin has not been studied in this regard. Furthermore, the metabolic pathways of oleandrin, mediated by phase I and phase II enzymes, need to be studied.

Different pharmacokinetic characteristics of drugs may be caused by polymorphisms in drug-metabolizing enzymes (DMEs) and efflux transporters (ETs), which lead to different efficacies and toxicities among patients (Bi et al., 2013; Dai et al., 2015; Wu et al., 2018). For example, the use of paclitaxel for chemotherapy in patients with the CYP3A4*22 mutation can lead to a significant increase in its neurotoxicity (de Graan et al., 2013). Oleandrin is a substrate transported by P-gp (Zhou et al., 2019). The expression level of P-gp affects its bioavailability and toxicity in the body. Hence, investigating DMEs and ETs can not only clarify the mechanism of action of oleandrin in the body (Bi et al., 2013), but it may also be useful in the clinical design of personalized therapies using oleandrin.

The pH also has a significant effect on the metabolism of CGs. Gault in 1981 showed that an increase in gastric acid secretion caused digoxin hydrolysis in the stomach, producing a large amount of digoxin aglycone, and an increase in metabolites excreted in the urine (Gault et al., 1981). Wang in 2007 reported that after simulating the stability of the human digestive tract fluid, periplocin was unstable in the fasting gastric juice (pH 1–3) and was hydrolyzed into periplogenin by hydrolysis of glycosidic bonds (Wang et al., 2007). Metabolites from phase I and phase II *in vivo* metabolism of oleandrin require further extensive characterization, as do their pharmacological contributions. The biotransformation of oleandrin by commonly used microbial strains should be systematically studied, related products obtained, and the enzyme systems involved in the transformation process understood. It may be inferred from the number of metabolites whether there are differences in the metabolic pathways of oleandrin metabolism in the body under the action of the intestinal flora of different species such as humans and rats. *In vitro* metabolism studies, the structure of oleandrin can be modified to study microbial metabolic processes. In the future, we can use the summary of the reported research studies and the WinNonlin pharmacokinetic software to construct pharmacokinetic models of oleandrin to aid in systematic research.

### 4.4 Excretion

Excretion is the process of transporting the parent drug or its metabolites out of the body through excretory or secretory organs, either under acidic, alkaline, or neutral conditions (Liu, 2016). There are many avenues for excretion, including the kidneys, digestive tract, lung, skin, saliva, and milk. The tissue distribution and elimination information is extremely important when a drug showing significant pharmacological effects has a low oral bioavailability because concentrations of the drug in tissues outweigh those in the blood (Jia et al., 2005; Mekjaruskul et al., 2012).

Oleandrin, oleandrigenin, oleandrin conjugates, and other metabolites are excreted in feces, with a small quantity excreted in urine (Hanske et al., 2009). Gastrointestinal excretion refers to the process by which drugs and their metabolites, after secretion into the bile, enter the intestinal cavity through to the common bile duct, and are excreted in the feces. A case describing a 25-year-old woman who ingested aqueous extracts of oleander leaves and died 4 h later, revealed that the concentration of oleandrin in the kidney, liver, and bile, was significantly higher than that in other organs or serum (Zhai et al., 2018). This indicates that the elimination of oleandrin is facilitated by the liver and kidneys of the person (Zhai et al., 2018). Ni in 2002 showed that after 24 h of oleandrin injection, 8% of oleandrin and its metabolitesis excreted in the urine (out of which the oleandrigenin content accounts for 4.4% and oleandrin content accounts for 1.9%), and 66% is excreted in the feces (where the oleandrin and oleandrigenin contents are equal) (Ni et al., 2002). Therefore, fecal excretion via bile is the main route of elimination for oleandrin, with most of the substances in feces being metabolites from intestinal bacteria (Ni et al., 2002).

EHC leads to a significant prolongation of the effect of the drug in pharmacodynamics; the drug circulates in the body, increasing retention time in the body, and improving the drug utilization efficiency. EHC also leads to slow elimination and slower excretion in the body (Liu, 2016), resulting in accumulation poisoning and even a life-threatening event. The significance of EHC on excretion depends on the rate of drug excretion in the bile. When the amount of bile excreted is large, EHC can prolong the action time of the drug. If EHC is blocked, excretion is accelerated, and the toxic side effects are reduced. The excretion rate of oleandrin and its metabolites and the influence of gut microbiota on the bioavailability and excretion of the glycoside are worth analyzing *in vivo*. Administration of radio-labeled drugs to animals provide the most definitive information on the routes of drug clearance. The administered radiolabeled formulation is usually prepared by mixing the non-radioactively labeled drug with the radiolabeled ones so that the total concentration of the drug and its metabolites could be quantified by determining the radioactivity of the radioactive tracer (Gao et al., 2014).

## 5 Toxicology

### 5.1 Poisoning Cases

Oleandrin is highly toxic and easily accumulated *in vivo*, which may lead to life-threatening intoxication (Guangyi.Yang et al., 2017). Renier in 2013 reported 30 cases of oleander toxicosis in 1995–2010 (Renier et al., 2013). In children, a single leaf may be lethal (Wasfi et al., 2008). *N. oleande*r can be lethal at the dose of 0.5 mg/kg for animals (Kanwal et al., 2020). Among all the poisoning cases that could be searched, it was found that the blood concentration range of oleander non-fatal cases was 1.1–7 ng/ml, and the blood concentration range of oleandrin was 9.8–82.857 ng/ml in fatal cases (summarized in Table 2). Hence, oleandrin is considered toxic at a concentration of approximately 1–2 ng/ml (Berrin et al., 2002; Prassas and Diamandis, 2008), and acutely fatal at a concentration of approximately 9.8–10 ng/ml (Ni et al., 2002; Manna et al., 2006). To date, accidental oleander intoxication has been reported in different species (shown in Figure 4A) including cattle (Ceci et al., 2020), cats (Meyer et al., 1993), dogs (Markov et al., 1999), chickens (Omidi et al., 2012), horses (Hughes et al., 2002), sheep (Adam et al., 2002; Aslani et al., 2004), goats (Barbosa et al., 2008), mice (Haeba et al., 2002; Abdou et al., 2019), rats (Akhtar et al., 2014), rabbits (Taheri et al., 2012), donkeys (Smith et al., 2003), and humans (Langford and Boor, 1996; Zhai et al., 2018). Although oleandrin has a wide range of pharmacological activities (shown in Figure 3A), its poor water solubility, narrow therapeutic window, and serious side effects have hindered its druggability, resulting in severe limitations in clinical application (Gao et al., 2020). However, no consensus or standard therapy has been proposed (Group, 1997). In recent years, some scholars have pointed out that the therapeutic index of oleandrin induces apoptosis in human cancer cells at a significantly lower dose than the toxic dose to humans (Newman et al., 2008). This indicates its feasibility in clinical therapy. Oleandrin was recognized as the active principal ingredient in PBI-05204 in Phase I and Phase II clinical trials for treating cancer (Wojtyna and Enseleit, 2004; Roth et al., 2020). Anvirzel is an aqueous extract of *N. oleander*; in as early as 2000, the FDA approved a Phase I clinical study using Anvirzel for patients with advanced solid tumors (Prassas and Diamandis, 2008). The therapeutic dose of oleandrin is close to the toxic dose; thus, its blood concentration must be monitored clinically. Oleandrin is of medical and toxicological interest. It is necessary to summarize the toxicological characteristics of oleandrin, which can provide the possibility for its broader clinical application.

**TABLE 2:** Summary of oleandrin concentration in biological liquids in reported intoxication cases of oleander in humans.

Case No	Age	Gender	Blood concentration (ng/ml)		Urine concentration (ng/ml)	Vitreous humor concentration (ng/ml)	Cerebrospinal fluid concentration (ng/ml)	Ref
			Non-fatal cases	Fatal cases	Fatal cases	Fatal cases	Fatal cases	
1	49	Female		9.8^a^			10.1	Arao, (2002)
2	49	Male with diabetes		10.0				Wasfi et al. (2008)
3	47	Female	1.6					Pietsch et al. (2005)
4	45	Female	1.1					Tracqui A, (1998)
5	NS	NS	7.0					Wang et al. (2000)
6	25	Female		65.5	254.0			Zhai et al. (2018)
7	71	Male		37.5	83.8	12.6		Carfora et al. (2021)
8	58	Female		82.9^a^	254.9			Azzalini et al. (2019)
				36.1				
9	42	Female		14.7				Al et al. (2010)

a Cardiac blood; NS, not specified.

### 5.2 Poisoning Symptoms

**FIGURE 5:**
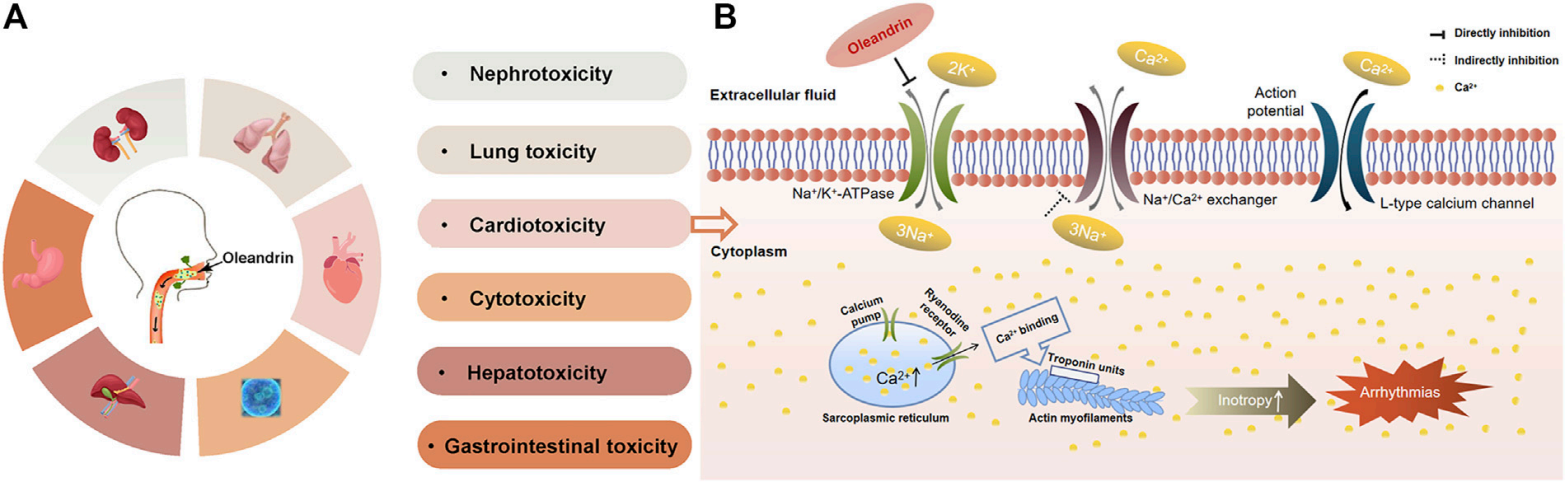
The toxicity of oleandrin and its typical toxicity mechanism. **(A)** Summary of the toxicity of oleandrin. **(B)** Schematic representation of oleandrin-mediated inhibition of Na/K-ATPase in cardiac myocytes.

As shown in Figure 5A, studies have demonstrated that oleandrin exposure results in injury of various organs, including the heart, liver, kidney, lung and gastrointestinal tract (Gao et al., 2020). Oleandrin is widely known for its typical dose-dependent cardiotoxicity, which inhibits Na/K-ATPase (Kucukdurmaz et al., 2012; Roberts et al., 2016; Botelho et al., 2020) causing electrochemical imbalance, which may damage the cardiovascular system even at low doses. Taheri in 2012 showed that interstitial pneumonia, cardiac muscle fiber degeneration, and necrosis occurred in sheep after ingesting oleander extract (Aslani et al., 2004). Mahendradhata in 2002 indicated that oleandrin may cause liver and kidney toxicity (Mahendradhata and Moerman, 2004). The liberation of free radicals during oleander metabolism may induce liver injury and disruption of hepatocyte membranes (Kwak et al., 2012; Taheri et al., 2012). Abdou in 2019 indicated that creatine kinase and creatine kinase-MB levels were elevated, indicating severe myocardial damage, confirmed by histopathological examinations (Abdou et al., 2019).

Symptoms of poisoning due to excessive intake of oleandrin are very similar to those of digoxin intake, in that the first symptoms commonly include gastrointestinal discomfort, nausea, vomiting, and abdominal pain, with mild to severe diarrhea (Chan and Buckley, 2014; Hudson et al., 2018; Botelho et al., 2020; Galton et al., 2020). These symptoms are followed by neurological symptoms, including drowsiness, insanity, generalized weakness, hyperkalemia, and potentially lethal cardiotoxicity, usually with arrhythmia, fibrillation by bradycardia and atrioventricular conduction block due to conduction issues (Clark et al., 1991; Haeba et al., 2002; Yanyan.; Cen et al., 2018; Pillay and Sasidharan, 2019; DiPietro and Mondie, 2021). The most common cardiac manifestation is atrioventricular junction blocks associated with increased ventricular automaticity. Secondary symptoms include bloody diarrhea, respiratory paralysis, burns to the mucous membranes of the eyes, mouth, and gastrointestinal tract, convulsions, and loss of consciousness (Alderslade and Davie, 1988).

### 5.3 Toxicity Mechanism

To date, the most accepted mechanism of oleandrin poisoning is the inhibition of Na/K-ATPase composed of a catalytic *a*-and glycosylated β-subunit (Weidemann, 2005; Bandara et al., 2010), a widely distributed membrane protein (as shown in Figure 5B). Na/K-ATPase pumps are attractive target for oncologists due to their versatile roles in signal transduction, maintenance of pH and induction of intracellular alkalinization (Mijatovic et al., 2007; Prassas and Diamandis, 2008). Na/K-ATPase inhibition leads to necrosis of myocytes since Na/K-ATPase is responsible for maintaining the membrane potential of the myocardial cells (Abdou et al., 2019). Na/K-ATPase uses energy derived from ATP hydrolysis to drive the active transport of K^+^ into the cells and Na^+^ out of the cells (Prassas and Diamandis, 2008). If this active transport is inhibited, an increase in intracellular Na^+^ and Ca^2+^ occurs, with a simultaneous reduction of intracellular K^+^. This results in an increase in cardiac contractile force and autonomy of heart cells, which slows conduction and causes arrhythmia (Qiu et al., 2010). Since oleandrin exhibits therapeutic and toxic effects by inhibiting Na/K-ATPase, further understanding of the positive inotropic effects of oleandrin established oleandrin as an effective drug for heart failure, which is still being used clinically. It is also well recognized that Na/K-ATPase-mediated signaling is involved in many physiological processes, including cell growth, differentiation, inflammation, muscle contractility, kidney function, and behavior (Lichtstein et al., 2018). Recent studies have highlighted a new aspect of the biology of Na/K-ATPase as a versatile signal transducer that binds CGs and activates multiple downstream signal transduction pathways (Geng et al., 2020). This implicates oleandrin in the regulation of many important physiological and pathological states (Xie and Askari, 2002; Kometiani et al., 2005; Aperia, 2007; Schoner and Scheiner-Bobis, 2007). Studies have shown that oleandrin regulates various signal transduction pathways, such as MAPK, NF-κB, and PI3K/Akt, which are often deregulated in cancer cells (Babula et al., 2013). In addition, Zhou in 2019 provided strong evidence that oleandrin is a substrate transported by P-gp, and that diurnal P-gp expression may be the main cause of oleander chronotoxicity in humans (Zhou et al., 2019).

### 5.4 Possible Ways to Decrease Toxicity

To expand the clinical application of oleandrin, methods need to be developed to reduce the toxicity of oleandrin based on the physicochemical properties, pharmacokinetic characteristics, and toxicological mechanisms in future research. Future clinical trials should not only take into consideration the dose, but also the formulations of oleandrin. Modern preparation technology and treatment strategies are effective methods to determine whether the toxicity of oleandrin can be reduced. Changing of *in vivo* dynamics and tissue distribution of the drug to exert attenuation and synergistic effects is also an effective strategy to try to solve the problem of the druggability of oleandrin. Recently, there have been many reports on the development of CG preparations, such as liposomes, micelles, and microspheres (Xiang et al., 2020). A series of explorations can be carried out on oleandrin with reference to the existing research on innovative CGs preparations. Studies have reported that oleander poisoning is ingestion time-dependent; mice and humans who ingested oleandrin during the middle and late of the light phase were more sensitive to oleander than when it was ingested at other times of the day (Caloni et al., 2012; Zhou et al., 2019). Personalized medicine is also considered to be a method for decreasing toxicity of oleandrin. By investigating gene polymorphisms, we can confirm whether personalized medicinal scheme of oleandrin is a novel strategy to decrease toxicity and increase efficacy. By referring to the research on digoxigenin, oleandrin can also be modified to achieve reduced toxicity, possibly through *in vivo* microbial metabolism (Jortani et al., 1996). Untangling the complex influence of the gut microbiome on oleandrin metabolism is challenging. Highly sensitive methods, such as untargeted MS-based metabolomics, are crucial tools necessary to uncover oleandrin-related mechanisms and pathways of metabolism (Tsunoda et al., 2021). Understanding the toxicity and side effects of drugs that undergo EHC will aid in the clinical use of existing drugs and the development of new drugs (Yu et al., 2019). The process of EHC can be adjusted according to the metabolism of the drug. If EHC is interrupted, such as through oral administration of the activated charcoal that binds the EHC drug to prevent it from reabsorption into the intestine, the half-life of the EHC drug will be decreased (Gao et al., 2014). Recent *in vitro* studies suggest that activated charcoal binds to oleander constituents effectively and provides gut decontamination (Shaw and Pearn, 1979). The new “-omics” research platforms can be employed to further uncover the toxicological mechanisms of oleandrin and determine the potential biomarkers of its toxic effects, aiding forensic and clinical medicine.

## 6 Conclusion

This review systematically summarizes the natural sources, structural characteristics, detection methods, pharmacokinetics (ADME), and toxicology of oleandrin. Oleandrin is a highly fat-soluble CG with broad pharmacological activities, mainly derived from *N. oleander*. However, oleandrin has a narrow therapeutic window and is highly toxic to multiple organs, and especially exhibits cardiotoxicity. LC-MS/MS is currently the most sensitive detection method for trace-level oleandrin in biological samples. The glycoside is rapidly absorbed, widely distributed, and has a low bioavailability *in vivo*. These phenomena may be related to the first-pass effect and P-gp efflux; however, further research is needed. Owing to its high fat solubility, oleandrin can easily pass through the BBB, enter the central tissues, and accumulate in tissues with a high fat content. It is mainly biotransformed by the liver and small intestine and it is easily hydrolyzed into oleandrigenin in the body. Other metabolic pathways have not been well characterized. The half-life of oleandrigenin is longer than that of oleandrin, and both are mainly excreted in the feces via bile. This suggests that oleandrin may have an EHC process. Pharmacokinetic research, especially in humans, is lacking; this restricts further development and utilization of oleandrin.

Seeking attenuation programs and improving the water solubility are key factors to improve the therapeutic capacity of oleandrin. The pharmacokinetic behavior and toxicity of oleandrin are reviewed, which can provide a systematic reference for the expansion of its clinical applications. A shorter pathway to clinical trials can be provided and the development of new drugs and clinically-safe medication can be facilitated if the toxicology and pharmacokinetics of oleandrin are well established. To date, oleandrin, as the main ingredient of several drugs, has been approved and used in clinical Phase I and Phase II trials. In the future, the possible requirement of personalized medicine-directed use of oleandrin for drug safety may be necessary and practical to achieve an ideal therapeutic efficacy with minimal toxicity in clinical practice. Currently, the world’s population is still plagued by COVID-19, generating much interest in antiviral drugs. The discovery of the novel antiviral activity of oleandrin will also accelerate future research, which may lead to the development and application of the glycoside in the treatment of various diseases.
